# MDP Up-Regulates the Gene Expression of Type I Interferons in Human Aortic Endothelial Cells 

**DOI:** 10.3390/molecules17043599

**Published:** 2012-03-23

**Authors:** Qingshan Lv, Mei Yang, Xueting Liu, Lina Zhou, Zhilin Xiao, Xiaobin Chen, Meifang Chen, Xiumei Xie, Jinyue Hu

**Affiliations:** 1 Department of Geriatric Cardiology, Xiangya Hospital, Central South University, Changsha 410008, China; Email: cathay057@sina.com (Q.L.); kyoym0131@yahoo.com.cn (M.Y.); xiaozl0223@163.com (Z.X.); chenxiaobinxy16@sina.com (X.C.); chenmeifang16@hotmail.com (M.C.); xyxiexm@sina.com (X.X.); 2 Central Laboratory, Renmin Hospital, Wuhan University, Wuhan 430060, China; Email: xueusually@163.com (X.L.); zhoulina1216@163.com (L.Z.)

**Keywords:** MDP, NOD2, interferon, endothelial cells, cytokine

## Abstract

Muramyldipeptide (MDP), the minimum essential structure responsible for the immuno-adjuvant activity of peptidoglycan, is recognized by intracellular nuclear-binding oligomerization domain 2 (NOD2). Here, we obtained evidence that the treatment of human aortic endothelial cells (HAECs) with MDP up-regulated the gene expression of type I interferons in a dose- and time-dependent manner. MDP also up-regulated the expression of the receptor *NOD2*, suggesting that MDP may induce a positive feedback response. The up-regulation of interferons was not dependent on the TNFα signaling, as HAECs did not express TNFα with the stimulation of MDP, and TNFα neutralizing antibody did not decrease the induction of IFNs induced by MDP. RT-PCR results showed that HAECs expressed the gene transcripts of interferon regulatory factor (IRF) 1, 2, 3, 9. The western blot results showed that MDP induced the phosphorylation of IRF3. These results suggested that MDP induced the up-regulation of gene transcript of interferons through the activation of IRF3 signaling pathway. Meanwhile, MDP induced the gene expression of pro-inflammatory cytokines, including IL-1β, IL-8, and MCP-1. Taken together, these results suggested that HAECs may play roles in the anti-infection immune response and in the induction of innate immunity.

## 1. Introduction

The conserved bacterial structures, called ‘pathogen-associated molecular patterns’ (PAMPs) are recognized by pattern recognition receptors (PRRs) to prime the activation of anti-bacterial host defense responses [1]. The best studied recognition pattern is lipopolysaccharide (LPS) of Gram-negative bacteria, which activates pattern recognition receptor toll-like receptor 4 (TLR4). To a certain extent, peptidoglycan (PGN) represents the counterpart to LPS in Gram-positive bacteria, which is recognized by TLR2 [1]. Muropeptides are breakdown products of peptidoglycan (PGN) of Gram-negative and Gram-positive bacteria. They are released during bacterial growth and division, as part of the host response by lysozyme and amidases, or upon antibiotic treatment. After phagocytosis of bacteria or bacterial breakdown products by host immune cells, the muropeptides trigger intracellular signaling cascades, leading to altered gene expression and activation of the immune response [2]. Muramyldipeptide (MDP) is the minimal bioactive peptidoglycan motif common to all bacteria, the essential structure required for adjuvant activity in vaccines. MDP has been shown to be recognized by a cytoplasmic PRP nucleotide-binding oligomerization domain 2 (NOD2), but not TLR2, nor TLR2/1 or TLR2/6 associations [2]. 

MDP has been reported to directly induce cytokines, to activate and modulate immune responses and inflammation. MDP was found to be related to the pathogenesis of periodontal disease by the induction of proinflammatory cytokines in human gingival fibroblasts [3]. NOD2 activation resulted in the production of IFN-gamma within the eye. Deficiency in IFN-gamma diminished the development of MDP-induced uveitis, indicating the crucial role of MDP in downstream inflammatory events [4]. In the bronchial epithelial cells, MDP and NOD2 play an important role for CXCL-8 release following LPS-challenge via synergistic interactions between MDP and LPS [5]. The murine bEnd.3 endothelial cells have been reported to induce a Th17 polarization following MDP stimulation [6]. In addition, MDP was found to protect mice from the development of experimental colitis by down-regulating multiple TLR responses [7]. 

In our study, we found that the activation of human aortic endothelial cells (HAECs) by MDP induced the production of type I interferons, suggesting that MDP may be useful in the induction of anti-viral immune response.

## 2. Results and Discussion

### 2.1. MDP Induces the Gene Expression of Type I Interferons

**Figure 1:**
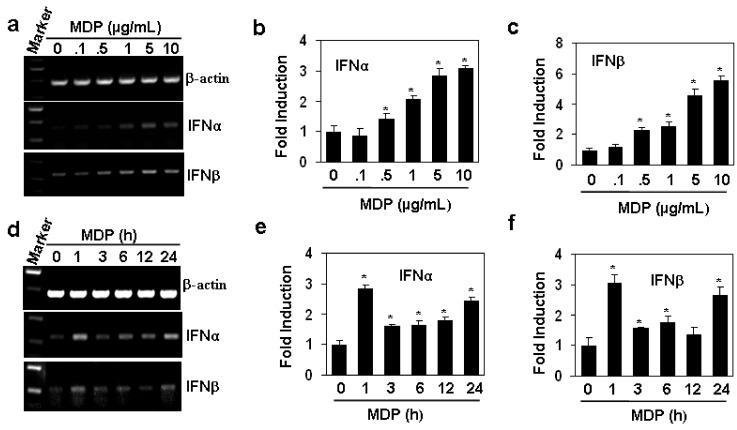
MDP induces the gene expression of type I interferons in human aortic endothelial cells. (**a**) MDP induced dose-dependent up-regulation of interferon gene expression. HAECs were treated with indicated concentrations of MDP for 24 h. The gene expression of IFNα and IFNβ was measured by RT-PCR. β-actin gene expression was measured as loading control; (**b,c**) The quantitation of interferon gene expression in (**a**) by qRT-PCR. *P < 0.05 compared with the control groups, n = 3 in each group; (**d**) MDP induced time-dependent up-regulation of interferon gene expression. HAECs were treated with 10 µg/mL MDP for indicated hours. The gene expression of IFNα and IFNβ was measured by RT-PCR. β-actin gene expression was measured as loading control; (**e,f**) The quantitation of interferon gene expression in (**d**) by qRT-PCR. * *P* < 0.05 compared with the control groups, n = 3 in each group.

TLR2, a receptor for the recognition of PGN, has been reported to induce the production of type I interferon responses [8,9]. In this study, we tested the effect of Nod2, another receptor of PGN, on the production of interferon-α and β with a specific ligand MDP in human aortic endothelial cells (HAEC). The results showed that the activation of NOD2 with MDP induced a dose dependent production of the gene transcripts of interferons (Figure 1a α and β induced by MDP with the concentrations from 0.5 to 10 µg/mL was significant (*P* < 0.05, Figure 1b,c). When the HAECs were treated with 10 µg/mL MDP for various hours, we found that the induction of interferons showed two peaks with one in 1 h and another in 24 h (Figure 1d–f). These results suggested that HAECs is a source of interferons by the activation of NOD2 with MDP.

### 2.2. The Effect of MDP on the Gene Expression of NOD1 and NOD2

**Figure 2:**
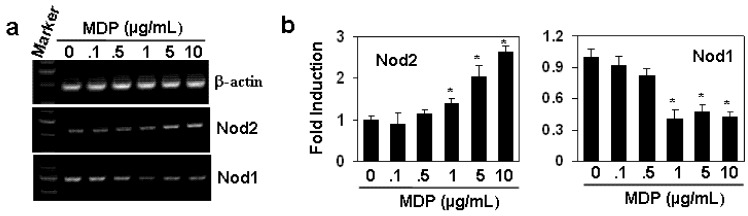
The effect of MDP on the gene expression of *NOD1* and *NOD2*. (**a**) HAECs were treated with indicated concentrations of MDP for 24 h. The gene expression of *NOD1* and *NOD2* was measured by RT-PCR. β-actin gene expression was measured as loading control; (**b**) The quantitation of *NOD1* and *NOD2* gene expression in (**a**) by qRT-PCR. *P < 0.05 compared with the control groups, n = 3 in each group.

The expression of NOD2 can be up-regulated through TLR4 in LPS-treated macrophages [10]. In our studies, we detected the effect of NOD2 ligand MDP on the expression of *NOD1* and *NOD2*. As shown in Figure 2a the treatment of HAECs with the various concentrations of MDP up-regulated the gene expression of *NOD2*, but down-regulated the gene expression of *NOD1*, a receptor for another breakdown product of PGN [11]. qRT-PCR results showed that the regulations induced by MDP were significant with the concentration of MDP from 1 to 10 µg/mL (Figure 2b). These results suggested that MDP has a positive feedback regulation for NOD2 expression. 

### 2.3. The Up-Regulation of Interferon Gene Expression is TNFα-independent

**Figure 3:**
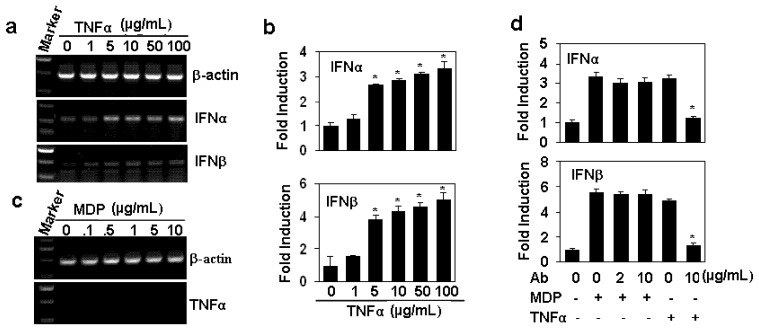
The up-regulation of the gene expression of interferons is TNFα-independent. (**a**) TNFα induced the up-regulation of interferon gene expression. HAECs were treated with indicated concentrations of TNFα for 24 h. The gene expression of IFNα and IFNβ was measured by RT-PCR. β-actin gene expression was measured as loading control; (**b**) The quantitation of interferon gene expression in (A) by qRT-PCR. * *P* < 0.05 compared with the control groups, n = 3 in each group; (**c**) The effect of MDP on TNFα expression. HAECs were treated with the indicated concentrations of MDP for 24 h. The gene expression of TNFα was measured by RT-PCR. β-actin gene expression was measured as loading control; (**d**) The effect of TNFα neutralizing antibody on MDP induced induction of interferon gene expression. HAECs, pre-treated with indicated concentrations of anti-TNFα neutralizing antibody for 30 min, were re-stimulated with 10 µg/mL MDP for 24 h. The gene expression of IFNs was measured by real-time RT-PCR. * *P* < 0.05 compared with TNFα-treated groups, n = 3 in each group.

Tumor necrosis factor (TNF) has been reported to induce the expression of interferon β through the activation of interferon-regulatory factor 1 (IRF1) pathway [12]. To confirm whether the up-regulation of interferons induced by MDP is dependent on the TNF pathway, we first detected the effect of TNFα on the gene expression of type I interferons in HAECs. As expected, the treatment of HAECs with the various concentrations of TNFα induced a dose-dependent up-regulation of interferon α and β (Figure 3a,b). However, when we tested the effect of MDP on TNFα expression, the results showed that no gene expression of TNFα was found within 24 h (Figure 3c. Furthermore, when HAECs were pre-treated with TNFα neutralizing antibody, the induction of IFNs induced by MDP re-stimulation was not decreased (Figure 3d). These results suggested that the up-regulation of interferons induced by MDP is not dependent on the TNF signaling.

### 2.4. MDP Induces the Phosphorylation of IFR3 in HAECs

**Figure 4:**
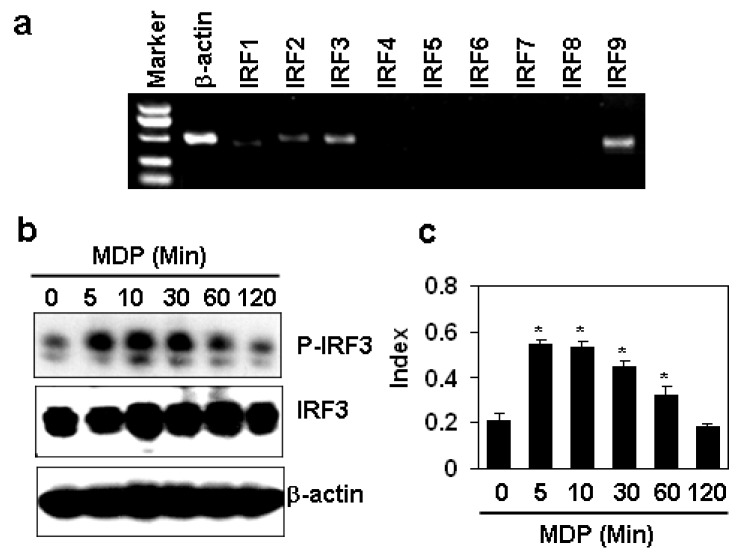
MDP induces the phosphorylation of IFR3 in HAECs. (**a**) The gene expression of IRFs in HAECs by RT-PCR; (**b**) The effect of MDP on the phosphorylation of IRF3. HAECs were treated with 10 µg/mL MDP for indicated minutes. The phosphorylated IRF3 was measured by Western blot. β-actin protein was measured as loading control; (**c**) The quantitation of protein levels of phophorylated IRF3 in (**b**). The levels of protein expression were semi-quantitated by scanning the pixel intensity of the bands of phophorylated IRF3 and normalized with the levels of total IRF3 proteins. * *P* < 0.05 compared with the control groups, n = 3 in each group.

The expression of type I interferons is regulated by the interferon regulatory factors (IRFs) in response to various factors [13,14,15]. To confirm which IRF is responsible for the induction of interferon α and β induced by MDP in HAECs, the IRF expression was first tested by RT-PCR. The results showed that HAECs expressed IRF1, 2, 3, 9, but not IRF4, 5, 6, 7, 8 (Figure 4a). Then we measured the phosphorylation of IRF3, which is the common regulator for interferon induction [13,14], and found that MDP induced the activation of IRF3 at time points 5, 10 and 30 minutes (Figure 4b). The semi-quantitation data showed the significant up-regulation of phosphorylated IRF3 compared with the non-treated group (Figure 4c). This data suggested that MDP may induce the up-regulation of type I interferons via IRF3 signaling pathway. 

### 2.5. MDP Induces the Gene Expression of Pro-Inflammatory Cytokines

**Figure 5:**
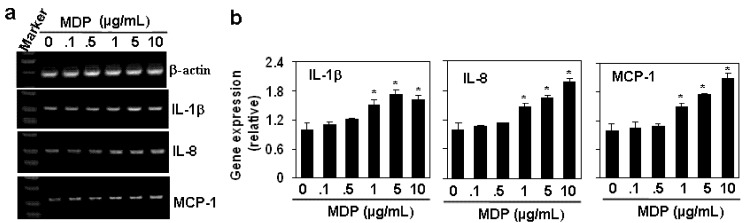
MDP induces the gene expression of pro-inflammatory cytokines. (**a**) The effect of MDP on the gene expression of pro-inflammatory cytokines. HAECs were treated with the indicated concentration of MDP for 24 h. The gene expression of pro-inflammatory cytokines was measured by RT-PCR. β-actin gene expression was measured as loading control; (**b**) The quantitation of cytokine gene expression in (**a**) by qRT-PCR. *P < 0.05 compared with the control groups, n = 3 in each group.

The activation of NOD2 by MDP induces the production of pro-inflammatory cytokines [16,17], and functions to regulate the innate immunity in response to infection [18]. The down-regulation of NOD2 reduces the MDP-induced cytokine production [19]. In our study, we also detected the effect of MDP on the pro-inflammatory cytokine expression in HAECs. The results showed that MDP induced dose-dependent up-regulation of pro-inflammatory cytokines, including IL-1β, IL-8 and MCP-1 (Figure 5a,b), suggesting that HAECs may also play roles in the induction of the innate immunity through the activation of NOD2. 

## 3. Experimental

### 3.1. Cell Lines and Reagents

Primary human aortic endothelial cells (HAECs) were purchased from ScienCell Research Laboratories (Carlsbad, CA, USA) and maintained in endothelial culture medium (ScienCell) supplemented with 5% FCS, 1% endothelial cell growth supplement, and antibiotics. All cells were cultured in a humidified atmosphere with 5% CO_2_ at 37 °C. Rabbit anti-human phosphorylated and total IRF3 antibodies were purchased from Cell Signaling Technology (Beverly, MA, USA). Muramyldipeptide (MDP), the minimal bioactive peptidoglycan motif and recognized by NOD2, was purchased from InvivoGen (San Diego, CA, USA). Recombinant human TNFα was purchased from ABGAB (Chicago, IL, USA). 

### 3.2. Reverse Transcription-PCR (RT-PCR)

Total RNA was extracted from 2 to 5 × 10^6^ cells using TRIzol (Invitrogen, Carlsbad, CA, USA), as described by the manufacturer. mRNA was reverse transcribed with RevertAid (MBI Fermentas, Burlington Ontario, Canada) at 42 °C for 60 min, and the resulting cDNA was subjected to PCR (95 °C for 1 min followed by 25–30 cycles at 95 °C for 30 s, 60 °C for 30 s, and 68 °C for 1 min and an extension for 10 min at 68 °C). PCR products were separated on 1.0% agarose gels and visualized with ethidium bromide. Forward and reverse primer pairs are listed (5´to 3´) as follows: 

**Table:** 

GAPDH-F,	AATCCCATCACCATCTTCCA,	GAPDH-R,	CCTGCTTCACCACCTTCTTG;
IFNα-F,	AGCTGCAAGTCAAGCTGCTCT,	IFNα-R,	TTCTTCACAGCCAAGATGGA;
IFNβ-F,	TCTCCTCCAAATTGCTCTCCT,	IFNβ-R,	TACTCCTTGGCCTTCAGGTAA;
IL-1β-F,	TTGAAGCTGATGGCCCTAAAC,	IL-1β-R,	CACCAAGCTTTTTTGCTGTG;
IL-6-F,	CCAGTACCCCCAGGAGAAGAT,	IL-6-R,	TTGCCTTTTTCTGCAGGAAC;
IL-8-F,	TTGGCAGCCTTCCTGATTT,	IL-8-R,	TCAAAAACTTCTCCACAACCC;
IRF1-F,	GAGATGATCTTCCAGATCCCA,	IRF1-A,	TGTAGCTGCTGTGGTCATCA;
IRF2-F,	GGTGGGATGTGGAAAAAGATG,	IRF2-A,	AAATGTCTGGCGGATTGGT;
IRF3-F,	AACCTGGAAGAGGAATTTCCG,	IRF3-A,	ATGGTCTGCTGGAAGACTTGG;
IRF4-F,	TGTGGGAGAACGAGGAGAAGA,	IRF4-A,	TCCAAACGTCATGGGACATT;
IRF5-F,	GGTCAACGGGGAAAAGAAA,	IRF5-A,	CCTGCACCAAAAGAGTAATCC;
IRF6-F,	TAAACGCTTCCAGATTCCCT,	IRF6-A,	TGATCCAGCTCATCTTCCTCA;
IRF7-F,	TGGCTCCTTGGAGAGATCA,	IRF7-A,	TTGGAGTCCAGCATGTGTGT;
IRF8-F,	ACGCTGGCAAGCAAGATTAT,	IRF8-A,	TCTGGGAGAATGCTGAATGGT;
IRF9-F,	CAGGATGCTGCCTTCTTCAA,	IRF9-A,	TGCTGCTCCCAATGTCTGAAT;
TNFα-F,	TGACAAGCCTGTAGCCCATGTT,	TNFα-R,	AGGGCAATGATCCCAAAGTAGA

### 3.3. Immunoblot

Endothelial cells (1–2 × 10^6^) were lysed in 200 mL lysis buffer (20 mM Tris, pH 7.5, 150 mM NaCl, 1% Triton X-100, 1 mM EDTA, 1 mM sodium pyrophosphate, 1 mM β-glycerophosphate, 1 mM Na_3_VO_4_, 1 mg/mL leupeptin). The cell lysate was centrifuged at 12,000 g at 4 °C for 5 min. Total proteins were electrophoresed on 10% SDS-PAGE gels, and transferred onto Immobilon P membranes (Millipore, Billerica, MA, USA). The membranes were blocked by incubation in 3% nonfat dry milk for 1 h at room temperature and then incubated with primary antibodies in PBS containing 0.01% Tween 20 overnight at 4 °C. After incubation with a horseradish peroxidase-conjugated secondary antibody, the protein bands were detected with SuperSignal chemiluminescent substrate-stable peroxide solution (Pierce Rockford, IL, USA) and BIOMAX-MR film (Eastman Kodak Co., Rochester, NY, USA). When necessary, the membranes were stripped with Restore Western blot stripping buffer (Pierce) and re-probed with antibodies against various cellular proteins.

### 3.4. Quantitative Real Time RT-PCR (qRT-PCR)

The qRT-PCR was performed as described by Sun *et al*. [20]. Briefly, total RNA from cells was isolated and reverse transcribed as above. The cDNA was amplified using TaqMan Universal PCR master mix (Applied Biosystems, Foster City, CA, USA) and an ABI Prism 7500 sequence detection system (Applied Biosystems). Amplification of the target genes was normalized using the amplification levels of glyceraldehyde-3-phosphate dehydrogenase (*GAPDH*) as an endogenous control. The efficiency of the PCR was tested by amplification of the target from serially diluted cDNA generated from the reverse transcription of a stock set of human RNA. Data analysis and calculations were performed using the 2^−ΔΔ^*^CT^* comparative method, as described by the manufacturer. Gene expression is shown as the fold inductions of a gene measured in MDP-treated samples, relative to samples cultured with medium.

### 3.5. Statistical Analysis

All experiments were performed at least three times, and the representative results are shown. Results are expressed as the mean ± S.D. Differences between groups were examined for statistical significance using Student’s *t * test, and *P* values equal to or less than 0.05 were considered statistically significant.

## 4. Conclusions

In our studies, MDP has been found to induce dose- and time-dependent production of type I interferons. The up-regulation of interferons is not dependent on the cytokine TNFα, but maybe dependent on the activation of IRF3 signaling pathway, as MDP induced the phosphrylation of IRF3. MDP also up-regulated the expression of *NOD2*, suggesting a positive feedback existed after MDP stimulation. In addition, MDP also up-regulated the pro-inflammatory cytokines, indicating that MDP may play roles not only in the anti-infection immune response, but also in the innate immunity.
